# Minireview: demystifying microbial reaction energetics

**DOI:** 10.1111/1462-2920.14778

**Published:** 2019-08-27

**Authors:** Jan P. Amend, Douglas E. LaRowe

**Affiliations:** ^1^ Department of Biological Sciences University of Southern California Los Angeles CA 90089 USA; ^2^ Department of Earth Sciences University of Southern California Los Angeles CA 90089 USA

## Abstract

The biology literature is rife with misleading information on how to quantify catabolic reaction energetics. The principal misconception is that the sign and value of the *standard* Gibbs energy ($\Delta G_{r}^{0}$) define the direction and energy yield of a reaction; they do not. $\Delta G_{r}^{0}$ is one part of the *actual* Gibbs energy of a reaction (*ΔG*_*r*_), with a second part accounting for deviations from the standard composition. It is also frequently assumed that $\Delta G_{r}^{0}$ applies only to 25 °C and 1 bar; it does not. $\Delta G_{r}^{0}$ is a function of temperature and pressure. Here, we review how to determine *ΔG*_*r*_ as a function of temperature, pressure and chemical composition for microbial catabolic reactions, including a discussion of the effects of ionic strength on *ΔG*_*r*_ and highlighting the large effects when multi‐valent ions are part of the reaction. We also calculate *ΔG*_*r*_ for five example catabolisms at specific environmental conditions: aerobic respiration of glucose in freshwater, anaerobic respiration of acetate in marine sediment, hydrogenotrophic methanogenesis in a laboratory batch reactor, anaerobic ammonia oxidation in a wastewater reactor and aerobic pyrite oxidation in acid mine drainage. These examples serve as templates to determine the energy yields of other catabolic reactions at environmentally relevant conditions.

## Introduction

Microbial catabolic reactions, in fact all chemical reactions, can only proceed if there is an energetic drive. In phototrophy, this drive is supplied by solar radiation. In chemotrophy, however, it stems from thermodynamic disequilibrium for the redox reaction of interest, and is commonly quantified with an expression of the change in the Gibbs energy of reaction (*ΔG*_*r*_). Unfortunately, misleading information on how to determine *ΔG*_*r*_ has been perpetuated in the environmental microbiology community through incomplete and, sometimes, incorrect explanations in the literature. The fundamental cause for this confusion resides in the difference between *ΔG*_*r*_ and $\Delta G_{r}^{0}$ or, stated differently, the difference between the *actual* Gibbs energy of a reaction and a hypothetical reference frame (or *standard state*). The same issues arise when expressing catabolic reaction energetics in terms of actual and standard redox (or electrode) potentials denoted as *E* and *E* ^0^ respectively (Amend and Teske, 2005). The purpose of this communication is not to identify the origin of this confusion, but to remedy it. The first step is to recognize its pervasiveness in the literature, including in many microbiology textbooks (e.g., Madsen, 2015; Madigan *et al*., 2018; Willey, 2020).

Another point of confusion relates to the definition of $\Delta G_{r}^{0}$. Contrary to countless proclamations in the literature (we again point to many widely read textbooks as the launchpad for this confusion), $\Delta G_{r}^{0}$ does not represent the Gibbs energy of reaction at 298.15 K (25 °C) and 1 bar (10^5^ Pa), with all reactants and products at concentrations of 1 molar (M) or 1 molal *(m)*. $\Delta G_{r}^{0}$ is, in fact, a function of temperature and pressure, and environmentally relevant temperatures and pressures can have substantial effects on the value of $\Delta G_{r}^{0}$ that must be taken into account. Furthermore, the standard states of reactants and products are expressed as activities, not concentrations; although activity and concentration are related, they are not the same thing. In this review, we hope to clear up some critical and common misconceptions about microbial reaction energetics, and then provide something akin to a ‘how‐to manual’ for determining values of *ΔG*_*r*_ (or *E*) for many relevant redox processes at physicochemical conditions that are commonly encountered in natural systems, impacted environments, or laboratory experiments.

## The value of $\Delta G_{r}^{0}$ is misleading

The value of $\Delta G_{r}^{0}$ is only one part of the total Gibbs energy yield of a reaction, *ΔG*_*r*_. The other part accounts for the chemical composition of the environment of interest (the *Q*‐term):

(1) $$\Delta G_{r}=\Delta G_{r}^{0}+\text{RTln}Q_{r}$$

where *R* and *T* stand for the gas constant and temperature (in K), respectively, and *Q*_*r*_ represents the activity product as defined below. Values of $\Delta G_{r}^{0}$ are typically calculated from those of $\Delta G_{i}^{0}$, which represent the *standard* Gibbs energies of formation from the elements for every reactant and product species (*i*) in the reaction (*r*). For detailed discussions, see Amend and Shock (2001) or LaRowe and Amend (2019b).

Values of $\Delta G_{i}^{0}$ (and therefore values of $\Delta G_{r}^{0}$) are functions of temperature and pressure. Using thermodynamic properties determined at 25 °C and 1 bar to describe microbial processes in, for example, the cold deep ocean or a hot spring system leads to unnecessary errors and sometimes flawed conclusions. Values of $\Delta G_{i}^{0}$ for a wide range of compounds can be found in countless thermodynamic data tables; most of these, however, are restricted to 25 °C and 1 bar. For more than four decades, efforts by several research groups have determined the necessary parameters to calculate the values of $\Delta G_{i}^{0}$ as a function of temperature and pressure for now more than 3000 compounds. For a recent compilation and detailed discussion of the approach, see Dick (2019) and references therein. To ease the calculation of microbial reaction energetics, Amend and Shock (2001) tabulated values of $\Delta G_{i}^{0}$ at 0–200 °C for >300 minerals, aqueous solutes and gases. All thermodynamic values provided in this review were calculated using SUPCRT92 (Johnson *et al*., 1992). The computer program CHNOSZ, available in the R environment, can also be used to carry out the thermodynamic calculations summarized below (Dick, 2019).

In addition to noting the critical difference between *ΔG*_*r*_ and $\Delta G_{r}^{0}$, we also remind the reader that it is essential to identify the phase (e.g., gas, aqueous, specific mineral) of each reactant and product in a reaction. To elucidate the importance of this point, let us consider gas (g) and aqueous (aq) versions of the same net catabolic process—hydrogenotrophic methanogenesis—where H_2_O refers to liquid water:

(2) $${\mathrm{CO}}_{2}\left(g\right)+4H_{2}\left(g\right)={\mathrm{CH}}_{4}\left(g\right)+2H_{2}O$$

and

(3) $${\mathrm{CO}}_{2}\left(\mathrm{aq}\right)+4H_{2}\left(\mathrm{aq}\right)={\mathrm{CH}}_{4}\left(\mathrm{aq}\right)+2H_{2}O$$

At 25 °C and 1 bar, $\Delta G_{2}^{0}$ = −130.4 kJ/mol and $\Delta G_{3}^{0}$ = −193.7 kJ/mol, a difference of 63.3 kJ/mol. Let us now also consider the effect of temperature on these reactions. At 85 °C and 1 bar, $\Delta G_{2}^{0}$ = −106.0 kJ/mol, a difference of 24.4 kJ/mol compared with its value at 25 °C. The 85 °C value for $\Delta G_{3}^{0}$ = −184.1 kJ/mol, a difference of 9.6 kJ/mol compared with its 25 °C value.

Some investigators prefer to write the hydrogenotrophic methanogenesis reaction with bicarbonate (HCO_3_^−^) in place of CO_2_

(4) $${{\mathrm{HCO}}_{3}}^{-}+H^{+}+4H_{2}\left(\mathrm{aq}\right)={\mathrm{CH}}_{4}\left(\mathrm{aq}\right)+3H_{2}O$$

At 25 °C and 1 bar, $\Delta G_{4}^{0}$ = −229.9 kJ/mol, a difference of 99.5 kJ/mol compared with the version written with gaseous CO_2_ (Reaction (2)) and a difference of 36.2 kJ/mol to the version with aqueous CO_2_ (Reaction (3)). If CO_2_(g), CO_2_(aq) and HCO_3_^−^ are in chemical equilibrium in a system, then *ΔG*_2_=*ΔG*_3_=*ΔG*_4_, even though, as just shown, $\Delta G_{2}^{0}$≠$\Delta G_{3}^{0}$≠$\Delta G_{4}^{0}$, where values differ by almost 100 kJ/mol. This is a clear example of why the *value* of $\Delta G_{r}^{0}$ is important (since it is a component of *ΔG*_*r*_), but is misleading on its own. To show that the *sign* of $\Delta G_{r}^{0}$ can also be misleading, we must first discuss the chemical composition of the system of interest.

## The chemical composition of the environment matters

The *Q*‐term in Equation (1) accounts for the chemical composition of the system of interest and, therefore, how different the environment is from the standard state. Its contribution to *ΔG*_*r*_ can be positive or negative and, depending on the environment and catabolic reaction, can exceed several hundred kJ/mol. In other words, ignoring the composition of the system by assuming concentrations of 1 M or 1 *m* for all reactants and products (as is often done) will lead to substantial—and entirely unnecessary—errors in energy calculations. [In bioenergetics, molality (*m*) is preferred, because a kg of water—as opposed to a litre of solution—is not affected by changes in ionic strength or density.] *Q*_*r*_, the activity product can be evaluated with the expression

(5) $$Q_{r}=\prod a_{i}^{\nu_{i}}$$

where *a*_*i*_ represents the activity of species *i*, and *ν*_*i*_ stands for its stoichiometric reaction coefficient. If we again consider methanogenesis (Reaction (3)) as an example,

(6) $$Q_{3}=\frac{a_{{\mathrm{CH}}_{4}\left(\mathrm{aq}\right)}∙a_{H_{2}O}^{2}}{a_{{\mathrm{CO}}_{2}\left(\mathrm{aq}\right)}∙a_{H_{2}\left(\mathrm{aq}\right)}^{4}}$$

Activities are related to, but certainly not equal to, concentrations. The activity of any species *i* can be determined with the relation

(7) $$a_{i}=\frac{C_{i}}{C_{i}^{0}}\gamma_{i}$$

where *C*_*i*_ stands for the concentration (usually in molal units), $C_{i}^{0}$ represents the standard state concentration (usually 1 *m*), and *γ*_*i*_ denotes the corresponding activity coefficient (which is unitless). In Table 1, we provide values of *γ*_*i*_ for uncharged aqueous species, cations (+1, +2, +3) and anions (−1, −2, −3) at temperatures from 0 to 100 °C and in solutions with ionic strengths (*I*) of 0.001–0.7 *m*. The numbers given in Table 1, calculated with the CHNOSZ program (Dick, 2019), may be slightly different from those of the relatively few individual aqueous species for which activity coefficients have been experimentally determined. They do, however, serve as very close estimates for all neutral and charged species of interest in catabolic reactions. For context, the ionic strength of most rivers and lakes is 0.001–0.005 *m*, and that of seawater is ~0.7 *m*. Note in Table 1 that, for uncharged species, *γ*_*i*_ is ~1, regardless of temperature or ionic strength, and thus *a*_*i*_ ≈ *C*_*i*_ (but unitless). Consequently, for Reaction (3),

(8) $$Q_{3}=\frac{a_{{\mathrm{CH}}_{4}\left(\mathrm{aq}\right)}∙a_{H_{2}O}^{2}}{a_{{\mathrm{CO}}_{2}\left(\mathrm{aq}\right)}∙a_{H_{2}\left(\mathrm{aq}\right)}^{4}}\approx \frac{\left[{\mathrm{CH}}_{4}\right]}{\left[{\mathrm{CO}}_{2}\right]{\left[H_{2}\right]}^{4}}$$

where [*i*] represents the concentration of *i* in the aqueous solution. Note that since the standard state for pure liquids (including water) is an activity of 1 and, in dilute aqueous solutions, the activity of water is very close to 1, [H_2_O] does not appear here.

**Table 1:** Individual ion and neutral species activity coefficients (unitless) as a function of ionic strength (*I*), species charge and temperature.

*I* = 0.001 *m*	Charge						
T (°C)	−3	−2	−1	0	+1	+2	+3
0	0.73	0.87	0.97	1.00	0.97	0.87	0.74
25	0.72	0.87	0.96	1.00	0.96	0.87	0.74
50	0.71	0.86	0.96	1.00	0.96	0.86	0.73
75	0.70	0.85	0.96	1.00	0.96	0.86	0.71
100	0.69	0.85	0.96	1.00	0.96	0.85	0.70

*I* = 0.01 *m*	Charge						
T (°C)	−3	−2	−1	0	+1	+2	+3
0	0.40	0.67	0.90	1.00	0.90	0.68	0.45
25	0.39	0.66	0.90	1.00	0.90	0.68	0.44
50	0.38	0.65	0.90	1.00	0.90	0.66	0.43
75	0.36	0.63	0.89	1.00	0.89	0.65	0.41
100	0.34	0.62	0.89	1.00	0.89	0.63	0.39

*I* = 0.1 *m*	Charge						
T (°C)	−3	−2	−1	0	+1	+2	+3
0	0.10	0.36	0.78	1.00	0.78	0.41	0.19
25	0.09	0.35	0.77	1.00	0.77	0.40	0.18
50	0.09	0.34	0.77	1.00	0.77	0.39	0.17
75	0.08	0.32	0.76	1.00	0.76	0.37	0.15
100	0.07	0.30	0.74	1.00	0.74	0.35	0.14

*I* = 0.7 *m*	Charge						
T (°C)	−3	−2	−1	0	+1	+2	+3
0	0.02	0.17	0.67	0.99	0.67	0.25	0.09
25	0.02	0.16	0.66	0.99	0.66	0.24	0.08
50	0.01	0.15	0.65	0.99	0.65	0.23	0.08
75	0.01	0.14	0.64	0.99	0.64	0.21	0.07
100	0.01	0.12	0.62	0.99	0.62	0.19	0.06

For ions, values of *γ*_*i*_ can be far from unity (see Table 1). This is especially true at elevated ionic strengths and for multivalent ions (e.g., SO_4_^2−^, Fe^2+^, Mn^2+^, PO_4_^3−^) even at low ionic strengths. For catabolic processes in seawater, a wastewater reactor, intracellular fluid or other elevated‐salinity solution using concentrations in place of activities will result in substantial, and again, unnecessary error. Consider, for example, sulphate in a marine system: the total average concentration of all aqueous sulphate‐bearing species is 2.8 × 10^−2^ *m*, but the corresponding activity of the SO_4_^2−^ ion is almost an order of magnitude lower (~2.9 × 10^−3^, see below). Calculating *ΔG*_*r*_ at 25 °C for sulphate reduction using total sulphate concentration in the place of the activity of SO_4_^2−^ leads to an error of ~6 kJ/mol. Of course, ignoring activity coefficients for the other species in the reaction will further compound the error.

When considering gases in catabolic reactions, activities (*a*_*i*_) are replaced with fugacities (*f*_*i*_). Fugacity of the *i*th gas is related to its partial pressure (*P*_*i*_) via a unitless fugacity coefficient (*λ*_*i*_), using

(9) $$f_{i}=P_{i}\;\lambda_{i}$$

[Note that *f*_*i*_ is formally in units of pressure, but for the *Q*‐term, it is rendered unitless by normalizing it with a reference value ($f_{i}^{0}$) of 1 bar.] In the abyssal ocean, the deep subsurface or any system with *in situ* pressures above ~50–100 bar, values of *λ*_*i*_ for most gases are <0.5 and can be <0.2. In those ecosystems, partial pressures must be converted into fugacities to obtain accurate values of *ΔG*_*r*_. However, in lower pressure environments, regardless of the temperature or ionic strength, values of *λ*_*i*_ are 0.99–1.00, and thus, very little error is introduced by equating *f*_*i*_ = *P*_*i*_. That is not to say, however, that the *Q*‐term can be ignored. On the contrary, and as shown below, it can contribute tens of kJ/mol to *ΔG*_*r*_ even in low‐pressure systems.

## The ‘biological standard state’ gets it only partially right

It has long been recognized that assuming all concentrations to be 1 *m* (or activities = 1) is problematic. To remedy this situation, the Interunion Commission on Biothermodynamics proposed the biological standard state, where pH is set to 7 (i.e., $a_{H^{+}}$ = 10^−7^), because the cytoplasm of most cells is circumneutral (Wadsö *et al*., 1976). This biological standard state is expressed as $\Delta G_{r}^{0\prime }$ for the Gibbs energy and as *E*^0′^ for the electrode potential of redox half‐reactions. In bioenergetic calculations, there are at least four potential pitfalls with the biological standard state. First, we now know many acidophiles and alkaliphiles with intracellular pHs far from 7. For example, in the hyperacidophilic thermophile *Picrophilus oshimae* that grows optimally at pH < 1, the intracellular pH is as low as 4.6 (van de Vossenberg *et al*., 1998). Second, pH 7 only corresponds to solution neutrality at 25 °C and 1 bar. At 2 °C, the temperature of much of the abyssal ocean, neutral pH is 7.4, and at 100 °C, where numerous hyperthermophilic archaea and bacteria live and thrive, neutral pH is 6.1. Third, and arguably the most important, the concentration (or activity) of every other reactant and product in the reaction of interest is still kept at 1.0 *m* in the biological standard state which, depending on the chemical species and the environment, can be off by many orders of magnitude. Fourth, the biological standard state typically specifies a temperature (usually 25 °C, but sometimes 37 °C), thereby perpetuating the myth that values of $\Delta G_{r}^{0}$ and $\Delta G_{r}^{0\prime }$ are not functions of temperature. In other words, the proton, whether as a reactant or product species, cannot be omitted from the *Q*‐term; it should be treated like any other chemical species in this regard. In fact, the energetics of most reactions can be very pH sensitive, because proton activities can easily vary over more than ten orders of magnitude in microbial environments of interest.

## Chemical speciation is often ignored; it should not be

**Figure 1:**
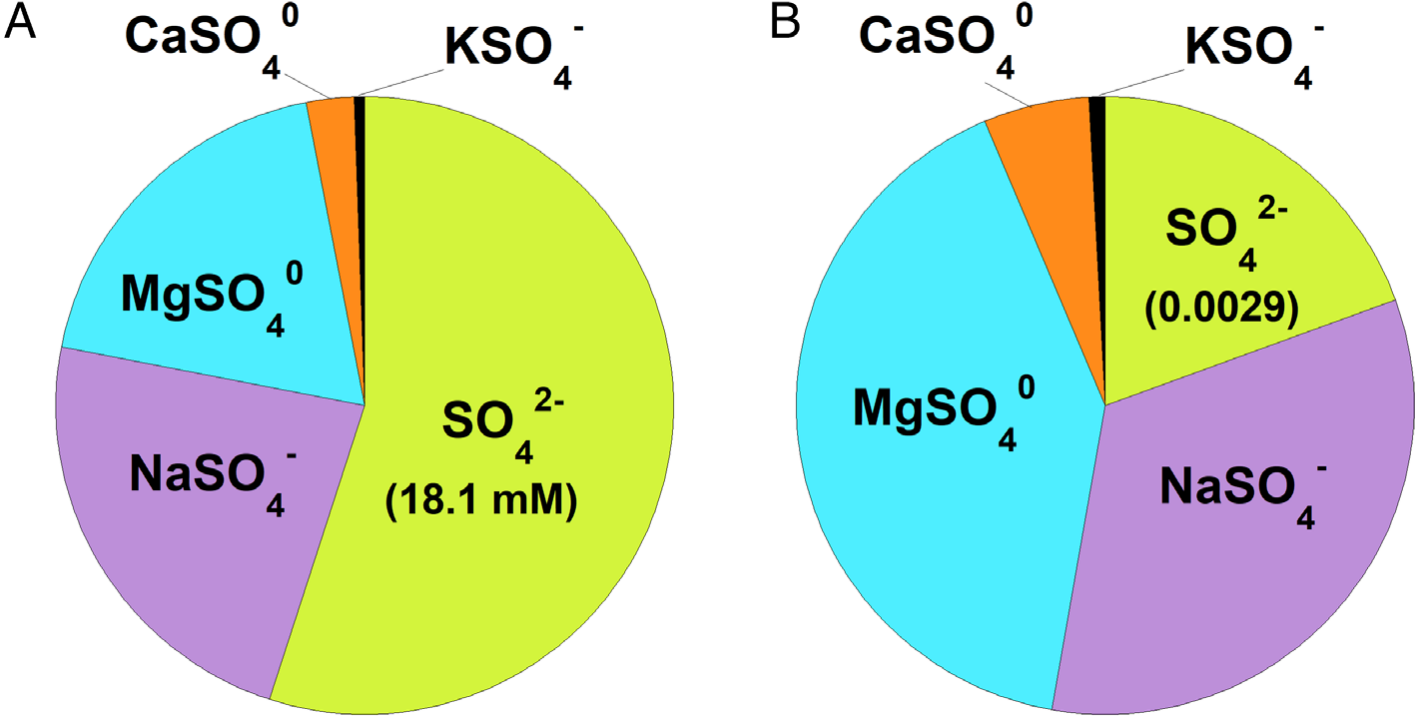
(A) Relative concentrations and (B) activities of the five most abundant sulphate‐bearing species in seawater at 25 °C and 1 bar for [SO_4_^2−^]_Total_ = 28 mM. The absolute concentration and activity of SO_4_^2−^ are also given. [Color figure can be viewed at http://wileyonlinelibrary.com]

All chemical reactions, including catabolic reactions, must be written in terms of chemical species. In determining reaction energetics, this is often—perhaps unknowingly—overlooked. Clearly, to calculate the values of $\Delta G_{r}^{0}$, we need to know the values of $\Delta G_{i}^{0}$ for all the species in the reaction as written. For the *Q*‐term, we need to know the corresponding activities (*a*_*i*_), which are generated from the concentrations (*C*_*i*_) and activity coefficients (*γ*_*i*_) as in Equation (7). However, most analytical methods used for aqueous solutions typically determine ‘total’ and not ‘species‐specific’ concentrations. For example, an analysis by ion chromatography may yield a total sulphate concentration ([SO_4_^2−^]_Total_), which is the sum of concentrations of all aqueous species containing the sulphate moiety (HSO_4_^−^, SO_4_^2−^, NaSO_4_^−^, MgSO_4_^0^, KSO_4_^−^, CaSO_4_^0^, and so on). As illustrated in Fig. 1A, for seawater with a total sulphate concentration of 28 mM, only 18.1 mM (or 65%) is as SO_4_^2−^; the rest is distributed among various inorganic complexes. Once the activity coefficient of SO_4_^2−^ ($\gamma_{{\mathrm{SO}}_{4}^{2-}}$ = 0.16 at 25 °C and 1 bar) is also taken into account, the activity of sulphate ($a_{{\mathrm{SO}}_{4}^{2-}}$) is nearly an order of magnitude less than its total concentration. It follows that SO_4_^2−^ is not the dominant sulphate‐bearing species in seawater; that label belongs to MgSO_4_^0^, followed by NaSO_4_^−^ (see Fig. 1B).

To accurately ‘speciate’ an aqueous solution can be rather involved and computationally intensive. It is accomplished by minimizing the Gibbs energy of the system for a given number of elements at a particular temperature, pressure and chemical composition. One also needs to know the values of $\Delta G_{i}^{0}$ for every species of interest (including complexes) at the *in situ* temperature and pressure. In all but the simplest cases, computer codes are typically used to determine rapid and accurate solutions to the Gibbs energy minimization problem. The most commonly used codes for such equilibrium speciation calculations include WATEQ (Truesdell and Jones, 1974; Ball *et al*., 1987), MINEQL (Westall *et al*., 1976; Schecher and McAvoy, 1998), EQ3 (Wolery, 1992), PHREEQC (Parkhurst and Appelo, 1999), and The Geochemists Workbench (Bethke, 2007). To be clear, the Gibbs energy minimization should not affect the redox state in the chemical speciation calculation. In other words, redox disequilibrium must be maintained to accurately assess the potential catabolic energy landscape. For a review of these and other software packages and the imbedded minimization techniques, see Leal *et al*. (2014).

It should be noted, however, that unless a system is of high ionic strength or the investigation centres on minor components (e.g., Cu‐, As‐, and Se‐redox processes), the most important speciation is often the pH‐dependence. Here, we'll use acetic acid as an example, and consider the two species CH_3_COOH(aq) and CH_3_COO^−^, related by the relation

(10) $${\mathrm{CH}}_{3}\text{COOH}\left(\mathrm{aq}\right)=H^{+}+{\mathrm{CH}}_{3}{\mathrm{COO}}^{-}$$

At pH < 4, [CH_3_COOH] >> [CH_3_COO^−^], and therefore, the concentration of ‘total’ acetate is essentially that of acetic acid. Conversely, at pH > 6, [CH_3_COO^−^] >> [CH_3_COOH], and ‘total’ acetate represents the concentration of CH_3_COO^−^. At 4 < pH < 6, the ‘total’ acetate must be speciated to avoid considerable and unnecessary error. Note that since chemical speciation is an equilibrium assessment, there is a temperature and pressure dependence that must be taken into account. For Reaction (10), however, this is relatively minor (Shock, 1995).

## The sign of $\Delta G_{r}^{0}$ can be misleading

If *ΔG*_*r*_ < 0, the reaction is exergonic. Whether $\Delta G_{r}^{0}$ < 0 is meaningless in this regard. Here, we provide two example reactions where the signs of *ΔG*_*r*_ and $\Delta G_{r}^{0}$ are, in fact, opposite. Consider sulphur disproportionation, which can be represented by

(11) $$4S^{0}+4H_{2}O=3H_{2}S\left(\mathrm{aq}\right)+{{\mathrm{SO}}_{4}}^{2-}+2H^{+}$$

where S^0^ denotes elemental sulphur with an activity of 1.0 (as for all pure minerals). At 25 °C and 1 bar, the *standard* Gibbs energy of this reactions ($\Delta G_{11}^{0}$) is 120.5 kJ/mol. Clearly, if the sign (and value) of $\Delta G_{r}^{0}$ was meaningful, this reaction, in the direction as written, would be impossible. However, this is a documented catabolism carried out by members of the Deltaproteobacteria, Thermodesulfobacteria and other bacterial phyla (Bak and Pfennig, 1987; Finster *et al*., 1998; Slobodkin *et al*., 2012; Kojima *et al*., 2016; Slobodkina *et al*., 2016). In environments with low levels of sulphide and sulphate and circumneutral to alkaline pH, the *Q*‐term for Reaction (11) has a large negative value that can counter the positive value of $\Delta G_{11}^{0}$, resulting in a net negative value of *ΔG*_11_. For example, at activities of 10^−6^ for H_2_S(aq) and SO_4_^2−^, and a pH of 7, Reaction (11) is strongly exergonic (*ΔG*_11_ = −96.4 kJ/mol). Similarly, values of $\Delta G_{r}^{0}$ are positive for most of the major fermentation reactions occurring in marine sediments (Canfield *et al*., 2005), but these reactions can be exergonic, because a negative *Q*‐term renders the overall Gibbs energy negative (LaRowe and Amend, 2019a).

Conversely, the sign of $\Delta G_{r}^{0}$ can be negative, but owing to unfavourable environmental conditions, that of *ΔG*_*r*_ can be positive—and therefore, the reaction would be endergonic. Here, we consider acetogenesis

(12) $$4H_{2}\left(\mathrm{aq}\right)+2{\mathrm{CO}}_{2}\left(\mathrm{aq}\right)={\mathrm{CH}}_{3}\text{COOH}\left(\mathrm{aq}\right)+2H_{2}O$$

At 25 °C and 1 bar, $\Delta G_{12}^{0}$ = −169.8 kJ/mol. Again, if the sign and value of $\Delta G_{r}^{0}$ were meaningful, this reaction should be energy‐yielding. However, in field or laboratory systems with relatively low levels of H_2_ and CO_2_, that is not the case. For example, at activities of H_2_ and CO_2_ equal to 10^−6^ and that of acetic acid equal to 10^−3^, this reaction is endergonic (energy‐consuming) with *ΔG*_12_ = 18.6 kJ/mol.

## Does *in situ* pressure matter?

At most conditions of interest in environmental microbiology, the effect of pressure on values of $\Delta G_{r}^{0}$ is secondary to that of temperature, and can often be ignored. Let us return to the methanogenesis examples described with 2, 3, 4. At 20 bar, corresponding to the average maximum depth (~200 m) of the photic zone in the ocean, values of $\Delta G_{2}^{0}$, $\Delta G_{3}^{0}$ and $\Delta G_{4}^{0}$ at 25 °C equal −130.3, −193.8 and −230.0 kJ/mol, respectively, differences of only 0.4, 0.1 and 0.1 kJ/mol compared with the low pressure (1 bar) values. At 350 bar, the approximate pressure at the average depth of the global ocean, values of $\Delta G_{2}^{0}$, $\Delta G_{3}^{0}$ and $\Delta G_{4}^{0}$ at 25°C are −129.2, −195.9 and −231.2 kJ/mol, respectively, differences that are only slightly more pronounced at 1.5, 2.2 and 1.3 kJ/mol compared to the 1 bar values. Pressure can, however, significantly affect the *Q*‐term (and hence *ΔG*_*r*_), especially if free or dissolved gases (e.g., O_2_, CO_2_, CH_4_, H_2_, H_2_S) are part of the target catabolism. Gas solubility can increase demonstrably with increasing pressure, resulting in higher concentration (and thus activity) of the corresponding aqueous solute. For example, if aqueous and gaseous H_2_ are in equilibrium at constant temperature (e.g., 25 °C), then the activity of H_2_ ($a_{H_{2}}$) can increase by several orders of magnitude from 7.85 × 10^−4^ at 1 bar to 1.54 × 10^−2^ at 20 bar to 1.93 × 10^−1^ at 350 bar.

## The Gibbs energy function that matters—calculating Δ*G*_*r*_

In the sections above, we discussed the wide‐ranging effects of temperature (and pressure) on $\Delta G_{r}^{0}$, as well as the wide‐ranging effects of chemical composition, including speciation, on the *Q*‐term. In Box 1, we work through five specific examples (A–E) on how to calculate values of *ΔG*_*r*_ (from values of $\Delta G_{r}^{0}$ and the *Q*‐term) for microbial catabolisms at defined environmental conditions. In light of the limited effects of pressure on $\Delta G_{r}^{0}$ and to permit comparisons, the energetics in these examples were determined at 1 bar. It should also be explicitly stated that the calculated values of *ΔG*_*r*_ apply only to the specified chemical composition. In open systems, it is assumed that these conditions are maintained. In closed systems (e.g., sealed bioreactors), the value of *ΔG*_*r*_ changes as the concentrations (and hence, activities) of reactants and products change with time. The chosen examples consider oxic and anoxic environments; freshwater and seawater; aqueous solutes, gases and minerals; organic and inorganic electron donors; natural, impacted, engineered and laboratory systems; and a range of temperatures. These examples are not intended to represent all, or even a majority, of environmental microbial catabolisms. However, each example can serve as a template to determine *ΔG*_*r*_ for similar processes under similar environmental conditions. The steps are:

1. Write a mass‐ and charge‐balanced chemical reaction, and identify the phases.
2. Determine the temperature and chemical composition of the system.
3. Speciate the aqueous solution as necessary.
4. Estimate activity and/or fugacity coefficients as necessary.
5. Obtain values of $\Delta G_{r}^{0}$ and the *Q*‐term to calculate *ΔG*_*r*_.

Box 1 **Calculating *ΔG*_*r*_ for five different microbial catabolisms under different environmental conditions.**

**Table:** 

A.Mesophilic aerobic respiration of glucose (C_6_H_12_O_6_) in a freshwater ecosystem
1. Glucose(aq) + 6O_2_(aq) = 6CO_2_(aq) + 6H_2_O(A)
2. 25 °C, *I* = 0.01 *m*, pH 7, [glucose] = 1 μ*m*, dissolved oxygen and dissolved inorganic carbon (DIC) at saturation with the atmosphere ([O_2_] = 259 μ*m*, [DIC] = [CO_2_] + [HCO_3_^−^] = 220 μ*m*).
3. Considering CO_2_(aq) + H_2_O = H^+^ + HCO_3_^−^, with $\Delta G_{r}^{0}$ = 36.22 kJ/mol and the corresponding equilibrium constant (*K*_*r*_) equal to 4.51 × 10^−7^, then $a_{{\mathrm{CO}}_{2}}$= 0.22 $a_{{\mathrm{HCO}}_{3}^{-}}$.
4. Activity coefficients (*γ*) from Table 1 for glucose (1.00), O_2_ (1.00), CO_2_ (1.00).
5. Using thermodynamic data at 25 °C, $\Delta G_{A}^{0}$ is −2922.3 kJ/mol. Using Equations 5, 7, and parameters given in Steps 2–4, *Q*_*A*_ is 0.884. *ΔG*_*A*_ calculated with Equation (1) is then −2917.6 kJ/mol.
B. Psychrophilic anaerobic respiration (with sulphate) of acetate in marine sediments
1. CH_3_COO^−^ + SO_4_^2−^ = 2HCO_3_^−^ + HS^−^(B)
2. 10 °C, *I* = 0.7 *m*, pH 8.1, [total sulphate] = 28 m*m*, [total acetate] = [DIC] = 10 m*m*, [total sulphide] = 1 μ*m*.
3. At these conditions, [SO_4_^2−^] = 18.1m*m*, [CH_3_COO^−^] = 7.7 m*m*, [HCO_3_^−^] = 6.1 m*m*, [HS^−^] ≈ [total sulphide].
4. Activity coefficients (*γ*) from Table 1 for SO_4_^2−^ (0.16), acetate^−^ (0.66), HCO_3_^−^ (0.66), HS^−^ (0.66).
5. Using thermodynamic data at 10 °C, $\Delta G_{B}^{0}$ is −45.8 kJ/mol. Using Equations 5, 7, and parameters given in Steps 2–4, *Q*_*B*_ is 10^–6.13^. *ΔG*_*B*_ calculated with Equation (1) is then equal to −79.0 kJ/mol.
C. Thermophilic methanogenesis in a 2‐phase (gas + aqueous) laboratory experiment
1. CO_2_(g) + 4H_2_(g) = CH_4_(aq) + 2H_2_O(C)
2. 85 °C, *I* = 0.01 *m*, pH 6.5, $P_{{\mathrm{CO}}_{2}}$ = 0.2 bar, $P_{H_{2}}$ = 0.8 bar, [CH_4_] = 1 μ*m*.
3. If $P_{{\mathrm{CO}}_{2}}$ and $P_{H_{2}}$ are maintained at 0.2 bar and 0.8 bar, respectively, speciation calculations are not necessary.
4. Fugacity coefficients (*λ*) for CO_2_ (1.00) and H_2_ (1.00) and activity coefficient (*γ*) for CH_4_ (1.00) interpolated from information in Table 1.
5. Using thermodynamic data at 85 °C, $\Delta G_{C}^{0}$ is −85.2 kJ/mol. Using Equations 5, 7 and parameters given in Steps 2–4, *Q*_*C*_ is 10^–4.91^. *ΔG*_*C*_ calculated with Equation (1) is then equal to −118.9 kJ/mol.
D. Mesophilic anaerobic ammonia oxidation (anammox) in a wastewater reactor
1. NH_4_^+^ + NO_2_^−^ = N_2_(g) + 2H_2_O(D)
2. 36°C, *I* = 0.5 *m*, pH 7, [total ammonia] = 7.1 m*m*, [total nitrite] = 1.8 m*m*, $P_{N_{2}}$ = 0.1 bar.
3. At these conditions, [NH_4_^+^] ≈ [total ammonia], [NO_2_^−^] ≈ [total nitrite].
4. Activity coefficients (*γ*) for NH_4_^+^ (0.69) and NO_2_^−^ (0.69) and fugacity coefficient (*λ*) for N_2_ (1.00) interpolated from information in Table 1.
5. Using thermodynamic data at 36 °C, $\Delta G_{D}^{0}$ is −364.2 kJ/mol. Using Equations 5, 7 and parameters given in Steps 2–4, *Q*_*D*_ is 10^4.26^. *ΔG*_*D*_ calculated with Equation (1) is then equal to −339.3 kJ/mol.
E. Mesophilic aerobic pyrite oxidation in acid mine drainage
1. FeS_2_(py) + 3.5O_2_(g) + H_2_O = Fe^2+^ + 2HSO_4_^−^(E)
2. 25 °C, *I* = 0.5 *m*, pH 1, dissolved oxygen at saturation with the atmosphere ([O_2_] = 259 μ*m*), [Fe^2+^] = 0.026 μ*m*, [HSO_4_^−^] = 0.149 μ*m*.
3. Considering HSO_4_^−^ = H^+^ + SO_4_^2−^, with $\Delta G_{r}^{0}$ = 11.30 kJ/mol and the corresponding equilibrium constant (*K*_*r*_) equal to 1.05 × 10^−2^, then $a_{{\mathrm{HSO}}_{4}^{-}}$ = 9.55 $a_{{\mathrm{SO}}_{4}^{2-}}$.
4. Activity coefficients (*γ*) for Fe^2+^ (0.28), HSO_4_^−^ (0.67) and fugacity coefficient (*λ*) for O_2_ (1.00) interpolated from information in Table 1.
5. Using thermodynamic data at 25 °C, $\Delta G_{E}^{0}$ is −1205.6 kJ/mol. Using Equations 5, 7 and parameters given in Steps 2–4, *Q*_*E*_ is 10^8.41^. *ΔG*_*E*_ calculated with Equation (1) is then equal to −1157.7 kJ/mol.

The first example (A) represents mesophilic aerobic respiration of glucose (C_6_H_12_O_6_) in a freshwater (low ionic strength) ecosystem. Six‐carbon sugars and their polymer parent materials are important electron donors for many heterotrophic microorganisms. To better understand biogeochemical processes in lakes, rivers, wetlands, soils, laboratory experiments and countless other natural and engineered systems, it may be necessary to determine the energetics of the oxidation of carbohydrates, proteinaceous materials and an array of simple to complex organic compounds with O_2_ as the terminal electron acceptor.

The second example (B) represents psychrophilic anaerobic respiration of acetate in marine sediments. The upper‐most layer of marine sediments is typically oxic, where aerobic respiration dominates. Below the oxic zone, sulphate becomes the most important terminal electron acceptor, responsible for much of the oxidation of organic carbon. For the vast majority of the global ocean, the seawater–sediment interface is cold (~2–4 °C), but with increasing depth and the accompanying geothermal gradient, the sediment temperature increases. Other oxidants to be considered in these anoxic environments include NO_3_^−^, Fe^III^‐minerals (e.g., haematite, goethite, ferrihydrite) and Mn^IV^‐minerals (e.g., pyrolusite).

The third example (C) represents thermophilic methanogenesis in a 2‐phase laboratory experiment. Because laboratory experiments allow for careful control, constant monitoring and wide‐ranging chemical analyses, determining energetics is relatively easy. Note also that it is common to slightly overpressure sealed culturing vessels if a gas phase is present. If, in the example described here, the total pressure was 3 bar ($P_{{\mathrm{CO}}_{2}}\;$= 0.6 bar, $P_{H_{2}}$ = 2.4 bar), then *ΔG*_15_ would equal −135.3 kJ/mol. As alluded to in the section above, this difference of 16.4 kJ/mol compared with the 1 bar value is due almost entirely to the change in the *Q*‐term; the difference in $\Delta G_{15}^{0}$ between 1 and 3 bar is trivial at <0.1 kJ/mol.

The fourth example (D) represents mesophilic anaerobic ammonia oxidation (i.e., anammox) in a wastewater reactor. The chemical compositions in engineered and impacted systems can depart significantly from those in natural environments, making speciation calculations particularly important. In addition, fluctuating temperatures and pHs, constant aeration, settling out of solid phases, changing water activities and other physicochemical factors must be taken into account to calculate *ΔG*_*r*_.

The fifth example (E) represents mesophilic aerobic pyrite oxidation in acid mine drainage. In many catabolisms, minerals can serve as electron acceptor or electron donor, in addition to providing a physical template. It is worth noting that different minerals with the same chemical formula have different values of $\Delta G_{i}^{0}$. For example, at 25 °C and 1 bar, values of $\Delta G_{i}^{0}$ for anhydrous iron oxyhydroxides such as goethite, lepidocrocite and 2‐line ferrihydrite (all FeOOH) are −491.6, −479.9 and − 465.3 kJ/mol respectively. As noted above, activities of pure minerals (and pure liquids) are typically set to 1.0, but those of amorphous solids (e.g., Mn^IV^‐oxyhydroxides, opaline silica, coal, kerogen), solid‐solutions (e.g., olivine) or complex liquids (petroleum, bitumen) are not.

## Conclusion

As environmental microbiologists, we are typically confronted with communities of catabolically diverse organisms in complex chemical systems. Some of these organisms may be well characterized, with isolates in culture collections and genomes fully sequenced, but many may be largely unknown, including and perhaps dominated by clades that have no cultured representatives (microbial dark matter) (Lloyd *et al*., 2018). The systems of interest may be natural and pristine, contaminated or engineered. To answer important research questions, investigations may be carried out *in situ*, or environmental samples may be used in controlled laboratory studies. To obtain robust and quantitative results, we can make use of field measurements, biomolecular data, laboratory experiments, analytical chemistry and numerical modelling. An often desirable but also often daunting task in environmental microbiology research is to explain findings in an energetics context.

In this review, we attempted to demystify microbial reaction energetics by clearing up some of the most common confusions and misconceptions. Topping that list are the ideas that: 1) a negative $\Delta G_{r}^{0}$ means the reaction is exergonic—it does not; 2) the value of $\Delta G_{r}^{0}$ represents the energy yield—it does not; and 3) $\Delta G_{r}^{0}$ applies to 25 °C, 1 bar, and concentrations of 1 M—it does not. $\Delta G_{r}^{0}$ is a temperature‐ and pressure‐dependent function that, together with the *Q*‐term that accounts for the chemical composition of the system, enables the calculation of *ΔG*_*r*_. The sign of *ΔG*_*r*_ informs on the direction in which the reaction is exergonic (or if it is in equilibrium, where *ΔG*_*r*_ = 0), and the value of *ΔG*_*r*_ quantifies the accompanying energy yield. Similarly, when considering redox half‐reactions, the same rules apply to values of *E* ^0^ and *E*. Finally, it is worth reiterating that *ΔG*_*r*_ < 0 does not mean that the corresponding reaction *will* occur, only that it *may* occur. A quantitative assessment of thermodynamically favourable redox reactions in an environment only provides a framework within which to better understand microbial metabolism.
